# Case Report: Teprotumumab treatment in an HIV-infected patient with refractory thyroid eye disease: clinical and quantitative MRI evidence

**DOI:** 10.3389/fimmu.2026.1911723

**Published:** 2026-08-19

**Authors:** Mingyue Zheng, Jiamin Cao, Feng Zhang, Wei Xiong

**Affiliations:** Department of Ophthalmology, The Third Xiangya Hospital, Central South University, Changsha, Hunan, China

**Keywords:** HIV infection, immune reconstitution inflammatory syndrome, quantitative magnetic resonance imaging, teprotumumab, thyroid eye disease

## Abstract

**Background:**

People living with human immunodeficiency virus (HIV) may develop Graves’ disease as a delayed autoimmune manifestation of immune reconstitution inflammatory syndrome following antiretroviral therapy. Thyroid eye disease (TED) is uncommon in this setting. Teprotumumab targets the insulin-like growth factor-1 receptor (IGF-1R) and inhibits orbital fibroblast activation; however, its use in patients with HIV-associated TED has not previously been reported.

**Case presentation:**

A 37-year-old man with HIV infection developed Graves’ hyperthyroidism and active moderate-to-severe TED five years after HIV diagnosis. Disease activity recurred after intravenous glucocorticoids and tocilizumab and persisted despite bilateral orbital decompression. At initial presentation, the CD4^+^ T-cell count was 229 cells/μL, the CD4/CD8 ratio was 0.48, and the thyrotropin receptor antibody level was elevated.

**Results:**

After three teprotumumab infusions, the clinical activity score decreased from 3/7 to 1/7 in both eyes. Proptosis decreased from 21 mm in the right eye and 22 mm in the left eye to 20 mm bilaterally, and extraocular motility and diplopia improved. Quantitative magnetic resonance imaging (MRI) showed reductions in medial rectus diameter and apparent diffusion coefficient values. Systemic immune recovery remained incomplete, but no opportunistic infections, HIV-related clinical deterioration, infusion reactions, or clinically evident hearing impairment were observed.

**Conclusion:**

This case suggests that teprotumumab may improve clinical and imaging outcomes in selected patients with refractory HIV-associated TED despite incomplete systemic immune recovery. The relative dissociation between orbital improvement and conventional systemic immune indices suggests that local IGF-1R-dependent mechanisms may contribute to treatment response. Further studies are required to determine long-term efficacy and safety.

## 1 Introduction

Individuals with human immunodeficiency virus (HIV) receiving antiretroviral therapy (ART) may develop Graves’ disease as a delayed autoimmune manifestation of immune reconstitution inflammatory syndrome (IRIS) (1, 2). Thyroid eye disease (TED), an immune-mediated orbital complication of Graves’ disease, has rarely been reported in people living with HIV, and its immunopathogenesis in the context of HIV-associated immune dysregulation remains poorly understood (1).

Current management of moderate-to-severe active TED primarily relies on systemic immunosuppression, including glucocorticoids and targeted biologic agents for inflammatory pathways (3, 4). However, in HIV-infected patients, additional immunosuppressive treatment may increase the risk of opportunistic infections, presenting treatment challenges. Previous studies have primarily focused on systemic immunomodulation, such as B-cell depletion or cytokine inhibition (3), but evidence regarding the efficacy and safety of these approaches in HIV-infected patients with TED remains limited, and objective assessments of local orbital disease activity are lacking. Teprotumumab is a monoclonal antibody targeting the insulin-like growth factor-1 receptor (IGF-1R) and exerts its therapeutic effects by inhibiting orbital fibroblast activation and tissue remodeling in TED (4). To our knowledge, teprotumumab has not previously been reported in patients with HIV-associated TED.

In the context of complex immune dysregulation, it remains unclear whether the therapeutic response of TED still depends on the restoration of systemic immunity, or if there are cases where orbital immune regulation is independent of the systemic immune status. This study reports a patient with HIV infection and refractory TED who received teprotumumab after an inadequate response to multiple lines of treatment. Despite persistent abnormal systemic immune indicators, the patient’s orbital inflammation and quantitative magnetic resonance imaging (MRI) parameters showed marked improvement. This case suggests that orbital inflammation may improve despite incomplete systemic immune recovery and that teprotumumab may be a treatment option for refractory TED occurring in the setting of HIV-associated immune reconstitution.

## 2 Case presentation

**Figure 1:**
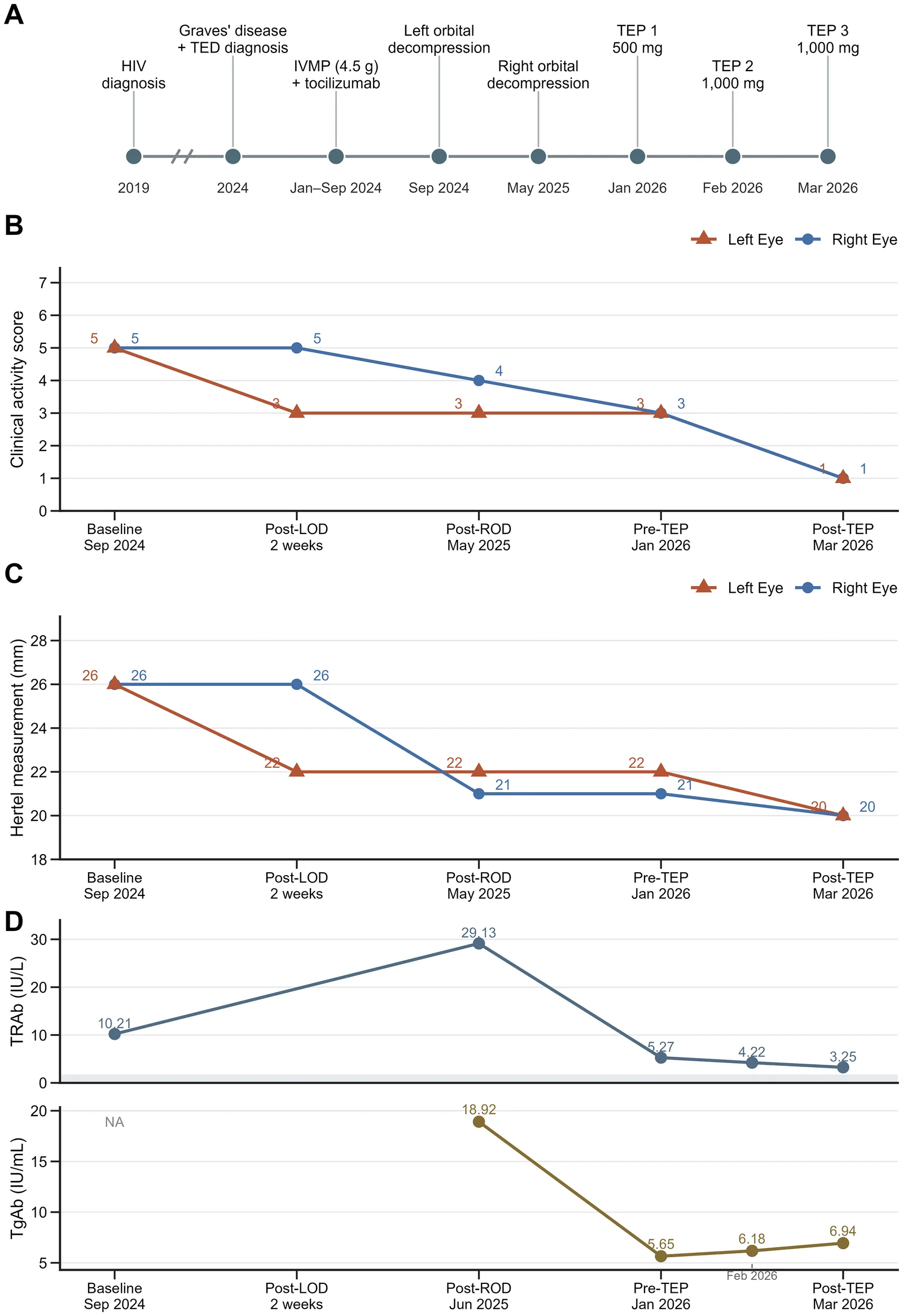
Clinical course and treatment response to teprotumumab in an HIV-positive patient with refractory thyroid eye disease. **(A)** Chronological timeline showing HIV diagnosis in 2019, Graves’ hyperthyroidism and TED diagnosis in early 2024, intravenous methylprednisolone and tocilizumab administered before referral, left orbital decompression in September 2024, right orbital decompression in May 2025, and three teprotumumab infusions from January to March 2026. **(B)** Longitudinal changes in the clinical activity score (CAS) in the right and left eyes. **(C)** Longitudinal Hertel exophthalmometry measurements in the right and left eyes. **(D)** Longitudinal changes in thyrotropin receptor antibody (TRAb; upper panel) and thyroglobulin antibody (TgAb; lower panel) levels. The shaded band in the upper panel indicates the laboratory reference range for TRAb. Thyroid-stimulating immunoglobulin (TSI) was not measured, and TgAb was unavailable at initial presentation but remained within the laboratory reference range during follow-up. IVMP, intravenous methylprednisolone; LOD, left orbital decompression; ROD, right orbital decompression; TEP, teprotumumab; CAS, clinical activity score; TRAb, thyrotropin receptor antibody; TgAb, thyroglobulin antibody; TSI, thyroid-stimulating immunoglobulin.

The patient was diagnosed with HIV infection in 2019 and had since received combination antiretroviral therapy with bictegravir/emtricitabine/tenofovir alafenamide. In early 2024, Graves’ hyperthyroidism and TED were diagnosed at another hospital, five years after the diagnosis of HIV infection, and the patient received intravenous methylprednisolone at a cumulative dose of 4.5 g and tocilizumab. These treatments produced a partial but transient clinical response. However, active disease relapsed after treatment discontinuation, with worsening orbital inflammation and the development of exposure keratitis. In September 2024, the 37-year-old male presented with bilateral proptosis for over a year, accompanied by progressive worsening of conjunctival hyperemia, orbital discomfort, and periorbital edema over the preceding 6 months (**Figure 1**). At presentation, the patient was euthyroid while receiving methimazole, with a free triiodothyronine (FT3) level of 3.62 pmol/L, a free thyroxine (FT4) level of 14.67 pmol/L, and a thyroid-stimulating hormone (TSH) level of 0.43 μIU/mL. He was an active smoker and continued smoking throughout treatment and follow-up.

**Figure 2:**
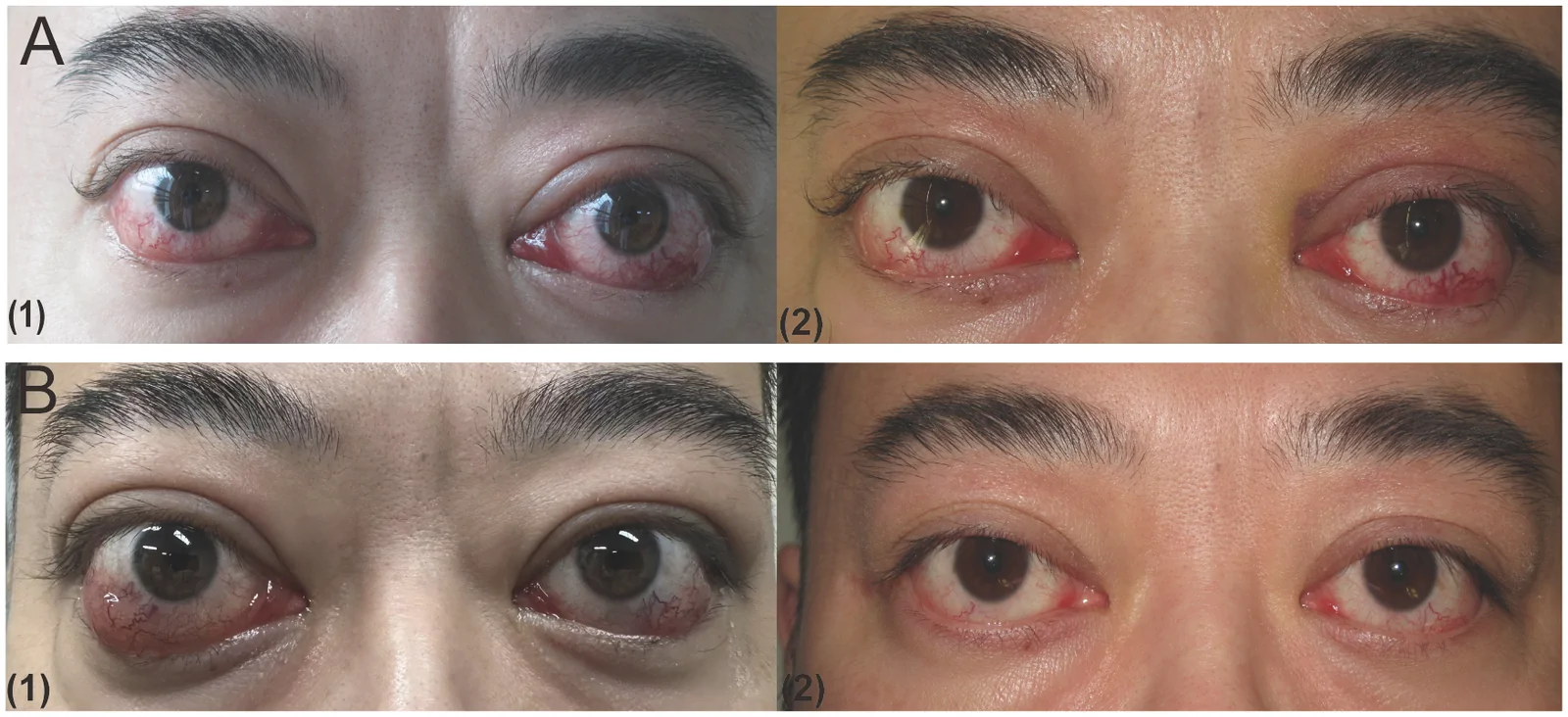
External ocular appearance before and after orbital decompression. **(A)** External appearance of both eyes, with particular attention to the left eye, before **(A1)** and 2 weeks after **(A2)** left orbital decompression. **(B)** External appearance of both eyes, with particular attention to the right eye, before **(B1)** and 2 weeks after **(B2)** right orbital decompression.

Ophthalmological examination in September 2024 revealed severe bilateral proptosis (26 mm in both eyes), moderate eyelid edema, eyelid retraction, and conjunctival congestion. Extraocular movements were severely restricted, particularly in vertical gaze (**Figure 2A1**), with diplopia on upward gaze. The clinical activity score (CAS) was 5 in both eyes.

Laboratory assessment demonstrated an elevated thyrotropin receptor antibody (TRAb) level of 10.21 IU/L. Thyroid-stimulating immunoglobulin (TSI) was not measured, and thyroglobulin antibody (TgAb) was unavailable at presentation. Subsequent TgAb levels remained within the reference range and are presented longitudinally in **Figure 1D** and **Table 1**, whereas thyroid peroxidase antibody (TPOAb) became elevated during follow-up.

**Table 1:** Changes in thyroid function and autoantibody profiles from baseline to the last follow-up.

Time	FT3 (pmol/L)	FT4 (pmol/L)	TSH (μIU/mL)	TgAb (IU/mL)	TPOAb (IU/mL)	TRAb (IU/L)	Tg (ng/mL)
2024-09	3.62	14.67	0.43	—	—	10.21 ↑	—
2025-06	6.52	18.25	0.03 ↓	18.92	24.25	29.13 ↑	500 ↑
2026-01	5.27	15.60	0.08 ↓	5.65	65.30 ↑	5.27 ↑	485 ↑
2026-02	5.61	19.90	0.49	6.18	66.50 ↑	4.22 ↑	348 ↑
2026-03	4.48	17.30	0.46	6.94	58.30 ↑	3.25 ↑	261 ↑

“↑” indicates levels above the reference range; “↓” indicates levels below the reference range; the absence of a symbol indicates values within the normal range.

FT3, free triiodothyronine; FT4, free thyroxine; TSH, thyroid-stimulating hormone; TgAb, thyroglobulin antibody; TPOAb, thyroid peroxidase antibody; TRAb, thyrotropin receptor antibody; Tg, thyroglobulin.

Immunological assessment showed a decreased percentage of CD4^+^ T-cells (19.43%), an inverted CD4/CD8 ratio (0.48), and a decreased absolute CD4^+^ T-cell count (229 cells/μL), consistent with chronic HIV-related immune dysregulation (**Supplementary Table 1**). The HIV viral load at presentation was unavailable. The peripheral lymphocyte count was reduced, and the level of interleukin-6 (IL-6) was elevated (43.36 pg/mL). Based on the ophthalmic findings, elevated TRAb levels, and a history of Graves’ hyperthyroidism, the patient was diagnosed with active moderate-to-severe TED in the context of chronic HIV infection.

Given the relapse after a partial response to intravenous methylprednisolone and tocilizumab, together with persistent severe proptosis and exposure keratitis, the multidisciplinary team selected orbital decompression rather than repeating the same systemic regimen.

The patient underwent left orbital decompression in September 2024. Two weeks postoperatively, left proptosis decreased from 26 mm to 22 mm, and the left CAS improved from 5/7 to 3/7; in contrast, right-eye disease remained active (proptosis: 26 mm; CAS: 5/7). Eight months later, right orbital inflammation progressed, with persistent exposure keratitis and marked elevations in TRAb and thyroglobulin levels (**Table 1**). Consequently, right orbital decompression was performed in May 2025. Although postoperative outcomes included reduced exophthalmos (right: 21 mm; left: 22 mm) and modest improvement in periorbital inflammation (right CAS: 4/7; left CAS: 3/7), residual active disease persisted during follow-up: bilateral CAS remained elevated at 3/7, and restrictive extraocular motility deficits were unchanged.

After multidisciplinary discussion among ophthalmologists, endocrinologists, and infectious disease specialists, teprotumumab was initiated because active TED persisted despite intravenous methylprednisolone, tocilizumab, and bilateral orbital decompression. **Figure 1** summarizes the treatment course (**Figure 1A**), longitudinal changes in bilateral CAS (**Figure 1B**), bilateral Hertel exophthalmometry measurements (**Figure 1C**), and longitudinal TRAb and TgAb levels (**Figure 1D**).

Antiretroviral therapy was continued throughout treatment, and the patient was closely monitored for opportunistic infections and treatment-related adverse events. Although eight infusions were planned, treatment was discontinued after the third infusion because financial constraints and limited drug accessibility precluded completion of the standard regimen. The patient therefore received three infusions at 3-week intervals: 500 mg for the first infusion, followed by two 1,000-mg infusions.

## 3 Results

**Figure 3:**
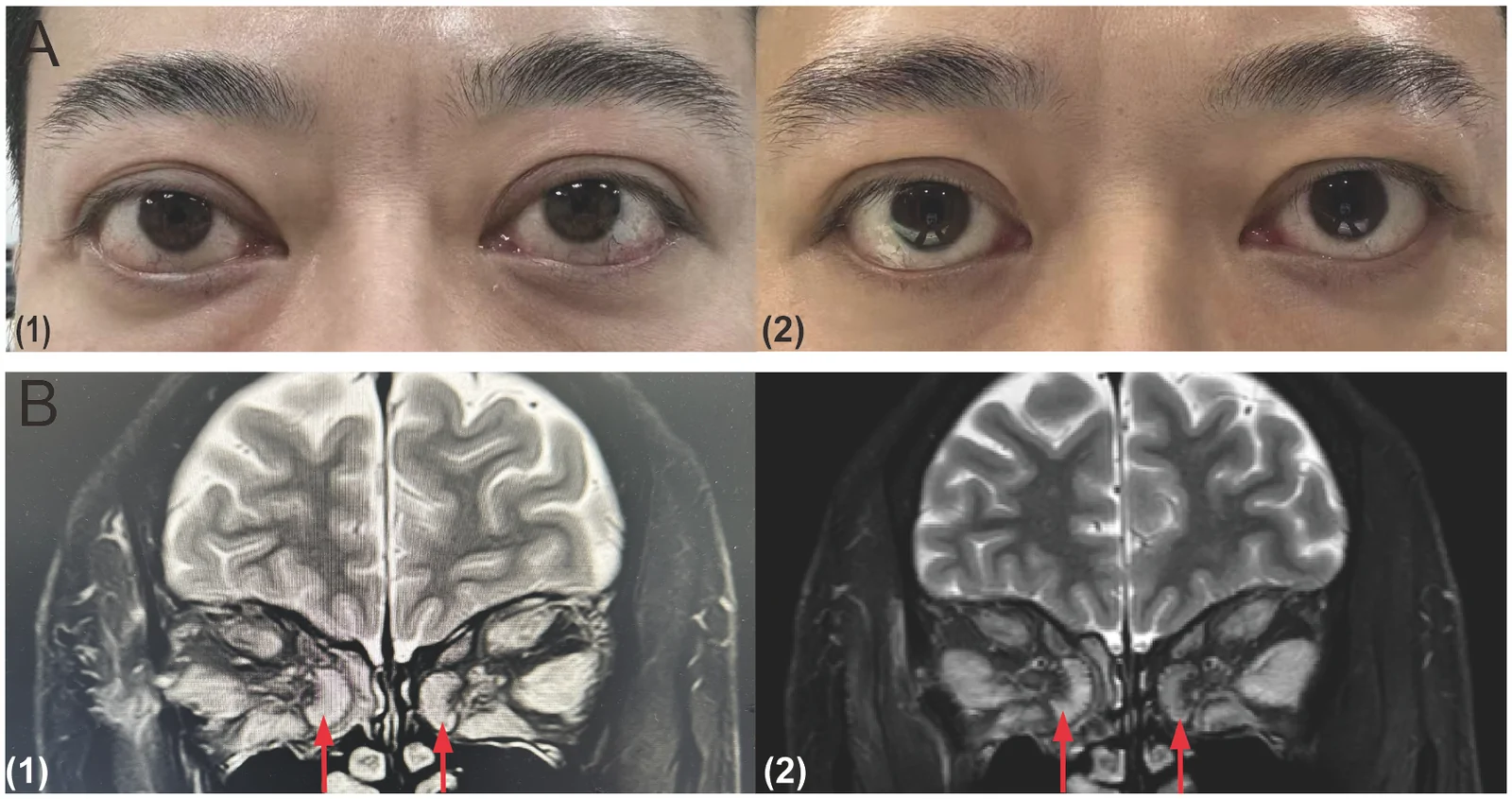
Clinical and radiological response to teprotumumab. **(A)** External ocular appearance before **(A1)** and after **(A2)** teprotumumab treatment. **(B)** Orbital MRI before **(B1)** and after **(B2)** teprotumumab treatment. Bilateral arrows indicate the medial rectus muscles and demonstrate reduced enlargement in both eyes after treatment.

At the final follow-up in March 2026, after three teprotumumab infusions, marked clinical improvement was observed, with substantial relief of ocular redness, periorbital swelling, eye pain, and photophobia (**Figure 3**). Immediately before teprotumumab treatment, after bilateral orbital decompression, Hertel exophthalmometry measurements were 21 mm in the right eye and 22 mm in the left eye; at the final follow-up, they had decreased to 20 mm in both eyes (**Figure 1C**). Extraocular motility improved markedly, particularly in vertical gaze, and the patient reported improvement in diplopia on upward gaze. No clinically evident ocular misalignment was observed in primary gaze. CAS scores decreased from 3/7 to 1/7 in both eyes, indicating transition to inactive disease (**Figure 1B**).

Quantitative orbital MRI findings were consistent with the observed clinical improvements (**Figure 3B**). The medial rectus diameter decreased from 6.67 mm to 6.11 mm, and the optic nerve apparent diffusion coefficient (ADC) decreased from 1.48 to 1.14 × 10^-^³ mm²/s. Visual comparison demonstrated reduced enlargement of the medial rectus muscles in both eyes after treatment, as indicated by bilateral arrows in **Figures 3B1**, **3B2**. ADC values of the extraocular muscles also decreased, and the complete quantitative MRI findings are summarized in **Table 2**. These reductions may reflect decreased tissue water content and inflammatory cell infiltration, as lower ADC values have been associated with reduced edema and inflammatory activity in TED (5).

**Table 2:** Changes in quantitative orbital magnetic resonance imaging parameters before and after teprotumumab treatment.

Parameter	Before teprotumumab	After teprotumumab
SRD (mm)	7.38	7.32
IRD (mm)	7.39	7.39
VMI	0.51	0.52
LRD (mm)	9.53	9.53
MRD (mm)	6.67	6.11
HMI	0.53	0.53
ON ADC (×10^-^³ mm²/s)	1.48	1.14
SR ADC (×10^-^³ mm²/s)	1.50	1.11
IR ADC (×10^-^³ mm²/s)	1.32	1.21
LR ADC (×10^-^³ mm²/s)	1.46	1.42
MR ADC (×10^-^³ mm²/s)	1.42	1.07

SRD, superior rectus diameter; IRD, inferior rectus diameter; VMI, vertical muscle index; LRD, lateral rectus diameter; MRD, medial rectus diameter; HMI, horizontal muscle index; ADC, apparent diffusion coefficient; ON, optic nerve; SR, superior rectus; IR, inferior rectus; LR, lateral rectus; MR, medial rectus.

During follow-up, complement component 3 (C3) increased from 0.69 g/L at baseline to 1.50 g/L in June 2025 and 1.49 g/L in February 2026, while complement component 4 (C4) increased from 0.09 g/L to 0.37 g/L and 0.25 g/L, respectively. Longitudinal analysis of lymphocyte subsets revealed that CD4^+^ T-cell counts increased from 229 cells/μL at baseline to 389–479 cells/μL during follow-up but remained below the laboratory reference range. The CD4/CD8 ratio increased from 0.48 to 0.86, entering the laboratory reference range during follow-up (**Supplementary Table 1**). Potential HIV-related ocular mimics were also assessed. Ophthalmic examination showed no clinical evidence of cytomegalovirus retinitis, and orbital MRI revealed no mass-like or infiltrative lesion suggestive of orbital lymphoma. No opportunistic infections or HIV-related clinical deterioration were identified during treatment or follow-up.

Pure-tone audiometry performed in March 2026 demonstrated normal bilateral hearing, with air-conduction pure-tone averages of 7 dB in the right ear and 15 dB in the left ear and speech recognition scores of 97% in both ears. Blood glucose remained normal throughout treatment. No infusion-related reactions or other clinically evident treatment-related adverse events were observed.

## 4 Discussion

Graves’ disease has been reported in approximately 1–2% of people with HIV as a late autoimmune manifestation of IRIS after ART (6); however, the incidence and prevalence of TED specifically attributable to IRIS remain unknown (7). Published evidence on HIV-associated TED is limited to small case series and individual case reports. In the Birmingham cohort, 11 patients with coexisting Graves’ disease and HIV infection were identified among 783 patients with Graves’ disease and 1,186 patients with HIV. Only three developed TED; all developed Graves’ disease more than 3 years after initiating highly active antiretroviral therapy, when their CD4^+^ T-cell counts had normalized and HIV viral loads were undetectable, and two required orbital decompression (1). In another reported case of IRIS-associated TED, treatment with methimazole and a short course of high-dose prednisone normalized thyroid function and eventually stabilized the ocular manifestations (6). Recovery of naive and recent thymic emigrant CD4^+^ T-cells after ART may permit the emergence of autoreactive lymphocyte clones and contribute to the development of Graves’ disease (2). Nevertheless, the mechanisms linking HIV-associated immune reconstitution and persistent immune dysregulation to TED remain unclear because of the limited number of reported cases.

Despite long-term ART, the persistently low CD4^+^ T-cell count in this patient may reflect incomplete immunologic recovery, sometimes described as immunologic non-response. Persistent immune activation, T-cell exhaustion, mitochondrial dysfunction, and impaired CD4^+^ T-cell homeostasis may contribute to inadequate CD4^+^ T-cell recovery despite ART (8, 9). However, the precise explanation in this patient could not be established because the pre-ART CD4^+^ T-cell nadir and longitudinal HIV viral-load data were unavailable. At presentation, the reduced CD4^+^ T-cell count and decreased complement levels indicated persistent abnormalities in both cellular and humoral immune parameters. Chronic HIV infection may contribute to persistent T- and B-cell dysfunction; however, the extent to which TED-related inflammation contributed to the complement abnormalities in this patient cannot be determined. Because HIV-associated TED has been reported only rarely, no standardized treatment strategy has been established; previously reported management has included antithyroid therapy, systemic corticosteroids, and orbital decompression, with variable clinical outcomes (1, 6).

Following teprotumumab treatment, marked clinical and imaging improvements were observed despite persistent systemic immune abnormalities. Throughout the follow-up period, the CD4^+^ T-cell count gradually increased but remained below the normal range. CAS, proptosis, extraocular motility, extraocular muscle thickness, and ADC values all improved after treatment. Because ART was continued throughout treatment, the modest increase in CD4^+^ T-cell count cannot be attributed to teprotumumab. Rather, the findings suggest that local orbital improvement may occur despite incomplete systemic immune reconstitution. The natural history of TED may include spontaneous reduction in inflammatory activity over time, as described by Rundle’s curve, although spontaneous improvement has been best documented in untreated mild disease (10). Smoking is a recognized modifiable risk factor for TED progression and severity, and smoking cessation is strongly recommended (11). In the present case, the patient continued smoking throughout treatment and follow-up. Therefore, spontaneous fluctuation in disease activity cannot be completely excluded when interpreting the observed response. Nevertheless, given the persistence of active disease despite glucocorticoids, tocilizumab, and bilateral orbital decompression, together with the continued exposure to smoking, the close temporal association between teprotumumab treatment and clinical and MRI improvement supports, but does not establish, a treatment effect.

Unlike glucocorticoids, rituximab, or tocilizumab, which mainly target systemic immune pathways, teprotumumab exerts its effect by inhibiting the IGF-1 receptor expressed on orbital fibroblasts. Activation of the IGF-1R pathway promotes cytokine production, adipogenesis, glycosaminoglycan accumulation, and tissue remodeling within the orbital region (3). However, IGF-1R expression is not restricted to orbital fibroblasts and has also been demonstrated on T and B lymphocytes. In Graves’ disease, increased IGF-1R expression has been reported on circulating and orbital T cells, where IGF-1R signaling may promote T-cell proliferation and survival (12). IGF-1R-positive B-cell populations are also expanded in Graves’ disease and may exhibit enhanced proliferation and immunoglobulin production (13). Therefore, teprotumumab may affect the interaction between local immune cells and fibroblasts in addition to directly suppressing orbital fibroblast activation.

An additional HIV-specific mechanism may also be relevant. Dubrovsky et al. demonstrated that extracellular vesicles carrying HIV-1 Nef induced long-lasting trained immunity and hyperreactivity in monocytes and macrophages. This effect was associated with increased lipid-raft abundance and enhanced cell-surface presentation of IGF-1R without increased *IGF1R* messenger RNA expression, suggesting post-transcriptional redistribution of functional IGF-1R to the cell membrane (14).

The proinflammatory phenotype was attenuated by pharmacologic inhibition of IGF-1R. These findings raise the possibility that persistent Nef-associated myeloid activation, even in the setting of suppressed HIV replication, may increase functional IGF-1R availability and contribute to chronic low-grade inflammation. Teprotumumab might therefore modulate not only orbital fibroblast activation but also IGF-1R-dependent interactions between immune cells and fibroblasts. However, this mechanism was demonstrated in experimental myeloid-cell models rather than orbital tissues or patients with TED, and its relevance to the present case remains speculative. The marked orbital improvement despite persistent lymphocyte-subset abnormalities is consistent with a relative dissociation between local orbital response and conventional systemic immune indices, but a single case cannot establish that these processes are biologically independent.

These findings highlight the need for clinical vigilance for Graves’ disease and TED in people with HIV undergoing immune reconstitution, particularly those with profound baseline immunosuppression. Quantitative MRI parameters (extraocular muscle thickness and ADC values) may serve as imaging biomarkers reflecting orbital inflammation and edema (15). However, their value for predicting response to teprotumumab requires validation in larger prospective studies with longitudinal imaging and immunologic follow-up.

In people with HIV, the safety assessment of teprotumumab should include surveillance for infection and monitoring for recognized adverse effects, particularly hyperglycemia and hearing dysfunction (4, 16). In this case, no opportunistic infections, infusion-related reactions, or HIV-related clinical deterioration were observed during treatment or follow-up, and pure-tone audiometry demonstrated normal bilateral hearing. These observations provide preliminary evidence of short-term clinical and imaging efficacy in a patient receiving continued ART, but they are insufficient to establish safety in the broader HIV population.

Although the standard eight-infusion regimen was initially planned, treatment was discontinued after the third infusion because limited drug accessibility and financial constraints prevented completion of the remaining doses. In addition, the absence of longitudinal HIV viral-load data limited assessment of the relationship between virologic control, immune recovery, and orbital treatment response. Larger studies incorporating complete teprotumumab regimens, longitudinal HIV viral-load measurements, systematic glucose and audiologic monitoring, and extended immunologic follow-up are needed to determine long-term efficacy and safety.

In conclusion, this case suggests that teprotumumab may produce meaningful clinical and imaging improvements in selected patients with refractory TED and HIV infection. The relative dissociation between persistent systemic immune abnormalities and marked orbital improvement suggests that local orbital mechanisms may contribute substantially to treatment response. Further research is required to clarify the immunologic mechanisms, immune-cell effects, and long-term safety of IGF-1R inhibition in this population.

## Data Availability

The original contributions presented in the study are included in the article/**Supplementary Material**. Further inquiries can be directed to the corresponding authors.
