# Supplementary material for: Case Report: Teprotumumab treatment in an HIV-infected patient with refractory thyroid eye disease: clinical and quantitative MRI evidence
Source: Front Immunol. 2026 Aug 19;17:1911723. doi: 10.3389/fimmu.2026.1911723 (PMC13534038; doi:10.3389/fimmu.2026.1911723)
Supplement: Supplementary file 1 [file SupplementaryFile1.docx]

**Supplementary Table 1 Longitudinal changes in peripheral T-cell subsets and selected immune parameters during follow-up**

| **Time** | **CD4⁺ T cells (%)** | **CD8⁺ T cells (%)** | **CD4/CD8 ratio** | **CD4⁺ T-cell count (cells/μL)** | **CD8⁺ T-cell count (cells/μL)** | **IL-6 (pg/mL)** | **IgG (g/L)** |
| --- | --- | --- | --- | --- | --- | --- | --- |
| Sep-24 | 19.43 | 40.23 | 0.48 | 229 | 472 | 43.36 | 7.05 |
| Jun-25 | 26.57 | 34.63 | 0.76 | 389 | 514 | 4.63 | 8.14 |
| Jan-26 | — | — | — | — | — | <2.3 | — |
| Feb-26 | 28.87 | 33.61 | 0.86 | 479 | 557 | — | — |

CD4, cluster of differentiation 4; CD8, cluster of differentiation 8; IL-6, interleukin-6; IgG, immunoglobulin G. Missing data are indicated by an em dash (—). Reference ranges were CD4⁺ T cells, 21–61%; CD8⁺ T cells, 12–47%; CD4/CD8 ratio, 0.71–2.78; absolute CD4⁺ T-cell count, 550-

1,440 cells/μL; absolute CD8⁺ T-cell count, 320–1,250 cells/μL; IL-6, ≤11.09 pg/mL; and IgG, 7.0–16.0 g/L.
